# Treating the invisible target: treatment planning targeting the microscopic tumor spread in gliomas

**DOI:** 10.3389/fonc.2026.1872089

**Published:** 2026-09-09

**Authors:** Wille Häger, Iuliana Toma-Dașu, Mehdi Astaraki, Marta Lazzeroni

**Affiliations:** 1 Department of Physics, Stockholm University, Stockholm, Sweden; 2 Department of Medical Physics, The Abdus Salam International Centre for Theoretical Physics, Trieste, Italy; 3 Department of Oncology and Pathology, Karolinska Institute, Stockholm, Sweden; 4 Department of Biomedical Engineering and Health Systems, Royal Institute of Technology, Huddinge, Sweden

**Keywords:** CTV, diffusion, dose painting, glioblastoma, glioma, treatment planning, tumor modelling, tumor spread

## Abstract

**Introduction:**

Glioblastoma (GBM) is the most common primary malignant brain tumor in adults, but treatment outcome is poor, and tumor recurrence is often unavoidable. Evidence suggests that regions with tumor cell densities below the medical imaging visibility threshold are more prevalent than the current treatment methodologies account for. This study aimed to explore different treatment planning strategies that account for the microscopic tumor cell distribution.

**Methods:**

A diffusion-proliferation model was used to predict the tumor spread in three patients with GBMs, each presenting different degrees of complexity. The tumor spread was quantified as the invasion volume *V_x_*, defined as the volume encompassed by a simulated cell density isocontour *x* (cells/mm^3^). Dose plans were created using targets defined by *V*_1000_, *V*_100_, and *V*_10_. Additionally, radiobiological modeling was used to predict the optimal dose per voxel needed to achieve a tumor control probability of 0.95 and treatment plans were made by mimicking the optimal dose distributions as reference. The difference between the reference dose distributions and mimicked dose plans was quantified as the percentage of voxels in the mimicked plan receiving a dose within 95% and 107% of the prescribed dose (quality index, *Q*_0.95–1.07_).

**Results:**

All dose plans generated using the first methodology were clinically acceptable in terms of tumor coverage and sparing of organs at risk. The mean quality index *Q*_0.95–1.07_, evaluated as a function of target volume across all cases, was 95% for the GTV, however, a decreasing trend as the target volume increased was observed.

**Discussion:**

Incorporating tumor spread models into treatment planning for GBM is feasible and warrants further investigation to potentially enhance treatment outcomes. Dose mimicking approaches based on reference dose distribution require careful planning and become particularly challenging for large, complex targets.

## Introduction

Glioblastoma (GBM) is a high-grade glioma well-known for its low overall survival rate. For patients undergoing standard treatment, the median overall survival time is less than 15 months and the 5-year survival is 5% (1). Despite being the most common primary malignant brain cancer (2), progress in improving treatment has been marginal, with the last breakthrough study published in 2005 by Stupp et al. (1) The Stupp protocol has been adopted as the standard treatment for GBMs and consists of surgical resection of the gross tumor (when possible), followed by radiotherapy with concomitant and adjuvant temozolomide.

Efficient treatment of GBMs remains a major clinical challenge. GBM tumors exhibit fast-growing rates with reported tumor volume doubling times as short as 5.3 days (3). Additionally, GBM cells exhibit highly invasive and diffuse infiltration into surrounding healthy brain tissue, complicating efforts to achieve complete local control (4–6). Histopathological investigations of GBM cases have revealed tumor cell invasion 4 cm away from the resected edge (4, 5), and that the spread appears anisotropic and largely undetectable from medical imaging (6). The high recurrence rate and low overall survival have been attributed to the far-reaching invasion of GBM cells into normal tissue. Localization of tumor cells relies on medical imaging. Imaging with MRI is frequently used for brain lesions, and many sequences are typically used, such as T1-weighted MRI (T1), T2-weighted MRI (T2), and fluid attenuated inversion recovery sequences (FLAIR). However, MRIs have a tumor cell density visibility threshold, which has been reported as 400 cells/mm^2^ (equivalent to 8000 cells/mm^3^) (7). Infiltrated tissue with tumor cell densities less than the visibility threshold will thus remain undetected, and may avoid irradiation if the infiltrated tissue is located outside the treatment targets. Correlation has been observed between recurrence site likelihood and vicinity to the resected volume edge, with increasing higher recurrence rates reported near the edge of the resected volume (8–11), suggesting that ensuring the observable target coverage might not be sufficient for local control.

The clinical target volume (CTV) is designed to encompass potential microscopic tumor cell infiltration beyond the gross tumor volume (GTV), accounting for tumor cells that are not detectable through conventional imaging. The CTV is conventionally defined by adding an isotropic margin around the GTV, constrained by anatomical barriers. Failure to improve treatment outcome has motivated the continual adjustment of the margin width throughout the development of modern radiotherapy. While the high rate of tumor recurrence suggests that the margin width should be increased to encompass more tumor cells, a landmark study by Shapiro et al. demonstrated improved survival with a 2 cm margin compared to whole-brain radiotherapy (12). This benefit was likely attributable to reduced toxicity and fewer complications in healthy brain tissue. Furthermore, evidence suggests that GBM tumor cells have greater motility in white matter than in gray matter (5, 13, 14), implying an anisotropic pattern of tumor spread that is poorly represented by conventional isotropic CTV expansions.

Recommended radiotherapy guidelines for GBM, as recommended by the European Organisation for Research and Treatment of Cancer (EORTC) (15), define the GTV as the combined volume of the surgical cavity and the contrast-enhancing tumor region visible on T1-weighted MRI. The CTV is defined by applying a 15 mm isotropic expansion to the GTV. The CTV should be trimmed at the natural barriers, such as the skull, the falx, the ventricles, organs at risk (OARs), and should not extend across hemispheres except through the corpus callosum. The planning target volume (PTV) is defined by expanding the CTV by 3 mm, while still adhering to the same anatomical restrictions as mentioned above. The recommended dose prescription is 60 Gy in 30 fractions to the PTV. Hypofractionated schedules may be used for elderly or frail patients. Depending on tumor location, relevant OARs may include the brainstem, Optic chiasm, optic nerves, eyeballs and lenses, and auditory nerves. Dose to the brain should be limited when possible, with one reported constraint limiting the volume receiving 60 Gy to no more than 3 cm^3^ (16).

The marginal improvement in treatment outcome for GBMs has led to the investigation of novel additions to the diagnostic and treatment strategies, such as tumor spread models which attempt to predict the direction and magnitude of tumor invasion into healthy tissue (17, 18). However, tumor spread models have not yet been integrated into clinical practice, primarily due to the challenges in model validation and the scarcity of clinical evidence. Moreover, recommendations on how such models may be implemented are even scarcer, making clinical adaptation difficult even when interest is present.

The aim of this study is to present a framework and proof-of-concept for GBM treatment planning that incorporates a tumor spread model to account for the predicted microscopic invasion into normal tissue, providing a foundation for outcome-driven, model-informed radiotherapy.

## Methods

A cohort consisting of 157 patients diagnosed with GBM was used in the study. The cohort originates from the Burdenko Neurosurgical Center, Moscow, Russia (19), in the form of medical images (CT, T1-MRI, T2-MRI, FLAIR MRI), delineated organs at risk (OARs), and follow-up status (treatment response, tumor progression). All image data was interpolated to a grid of 240 × 240 × 155 mm^3^ with a voxel size of 1 mm^3^. GTV structures were provided for only a subset of patients. For all remaining cases, an in-house GTV delineation approach was required. Due to the large size of the dataset, GTVs were delineated using an artificial intelligence (AI)-based methodology, as described by Häger et al. (20).

### Predicting the tumor spread

The tumor spread model used in this study has previously been investigated and thoroughly described by Häger et al. (20) and has been shown to correlate with overall survival (21), and treatment outcome (22). Briefly, the tumor spread was simulated using a mathematical diffusion-proliferation model for tumor infiltration of normal tissue (23, 24). The model describes the change in the number of tumor cells with time within a volume, which is assumed to be given by the summed effect of cell diffusion and cell proliferation:

(1) $$\frac{\partial c\left(r,t\right)}{\partial t}=\nabla \cdot D\left(\nabla c\left(r,t\right)\right)+\rho c\left(1-\frac{c\left(r,t\right)}{k}\right)$$

where *c*(**r**,t) is the tumor cell density (cells/mm^3^) as a function of coordinate r = r(x,y,z) and time *t*, D = D(r) is the diffusion coefficient (mm^2^) as a function of tissue present in **r**, *ρ* is the proliferation constant (1/day), and *k* is the combined biological and physical limitation of tumor cell count in normal tissue (cells/mm^3^). White, and gray matter tissue was segmented and assigned diffusion coefficients, *D_W_* and *D_G_*, respectively. Using Equation 1, tumor growth may be characterized by the ratios *D*/*ρ* and *D_W_*/*D_G_*, affecting diffusivity and anisotropy, respectively.

To simulate tumor spread for a given case, the GTV was used as a basis. With assumed homogenous tissue density and unknown biological tumor origin, the origin of the tumor cell distribution was set in one 1 mm^3^ voxel, located at the geometrical center-of-mass of the GTV. Tumor spread was simulated until the cell density isocontour of 8000 cells/mm^3^, assumed to be the visibility threshold, completely encompassed the GTV. Consequently, the GTVs derived from the simulated tumors (GTV_sim_) were defined as the volumes enclosed by the 8000 cells/mm^3^ cell density isocontours. A set of 30 different simulations were conducted, for each case, covering an input parameter range of *D*/*ρ* ∈ [0.1; 30.0] mm^2^ and *D_W_*/*D_G_* ∈ [10; 1000]. All obtained parameter-specific GTV_sim_ were compared to the true GTV segmented based on the diagnostic and planning images. The actual tumor spread was then assumed to correspond to the simulated tumor cell distribution obtained when using the input parameters that resulted in the closest match between GTV_sim_ and the GTV. Simulations were performed by discretizing Equation 1 and iterating using a finite-difference time-domain method. The simulations were conducted using MatLab v. 2012a. Using an Intel^®^ Core™ i3-3240 processor, computational times ranged between approximately 2 minutes to 2 hours, depending on whether the input parameters represented slow- or fast-growing tumors.

**Figure 1:**
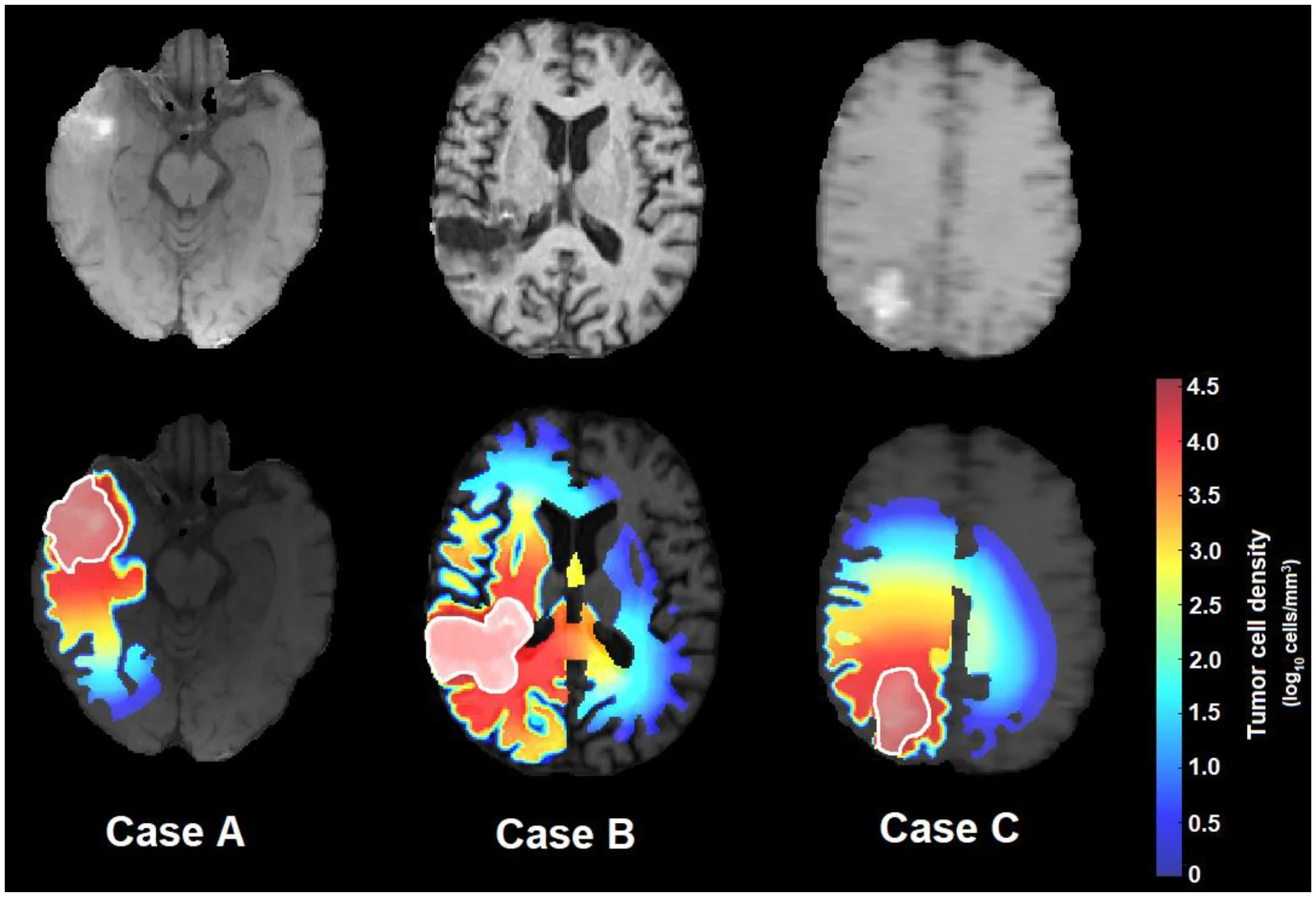
Three cases selected for treatment planning. The top row shows the raw T1 contrast enhanced images. The bottom row shows the segmented gross tumor volumes (encompassed by the white contours) and the modeled tumor spread is indicated by the colorwash. Case A exhibits a tumor in the anterior temporal lobe with moderate but anisotropic tumor spread. The tumor is situated near the brainstem. Case B exhibits a tumor in the inferior parietal lobe, with considerable tumor spread into the other hemisphere via the corpus callosum. Case C exhibits a tumor in the superior parietal lobe.

Three cases among the simulated ones were selected for subsequent treatment planning. They were chosen as representative candidates for planning based on tumor cell infiltration based on (a) good conformity of GTV_sim_ and the GTV, (b) confirmed post-treatment recurrence indicating unsatisfactory treatment outcome, and (c) variation in tumor location in the brain and in the extent of GBM-invaded brain tissue (**Figure 1**), thus posing distinct challenges for treatment planning.

### Treatment planning

All treatment plans and dose plan optimization were performed using a research version of RayStation v. 10B (RaySearch Laboratories, Stockholm, Sweden). Patient CT series were used for planning, along with the segmented GTVs and clinically delineated radiotherapeutic structures, including the OARs. All plans used a beam set of two arcs tailored to the target location (clockwise and counterclockwise, collimator angle offset 45°) of multi leaf collimated volumetric modulated arc therapy with 6 MV photon beams. The optimization tolerance was 10^-7^ and the optimizer was iterated until an optimal solution was found.

#### Methodology I: anisotropic CTV delineation

Three targets were defined in this study in addition to the GTV: CTV_1000_, CTV_100_, and CTV_10_, corresponding the volumes encompassed by the cell density isocontour of 1000 cells/mm^3^, 100 cells/mm^3^, and 10 cells/mm^3^, respectively, where a lower cell density isocontour implies larger target volume. From these CTVs, respective PTVs were defined by expanding the CTVs by 3 mm (PTV_1000_, PTV_100_, PTV_10_). The PTVs were administered 60 Gy over 30 fractions according to the EORTC recommendations and clinical goals were evaluated in terms of dose coverage to the PTVs.

**Figure 2:**
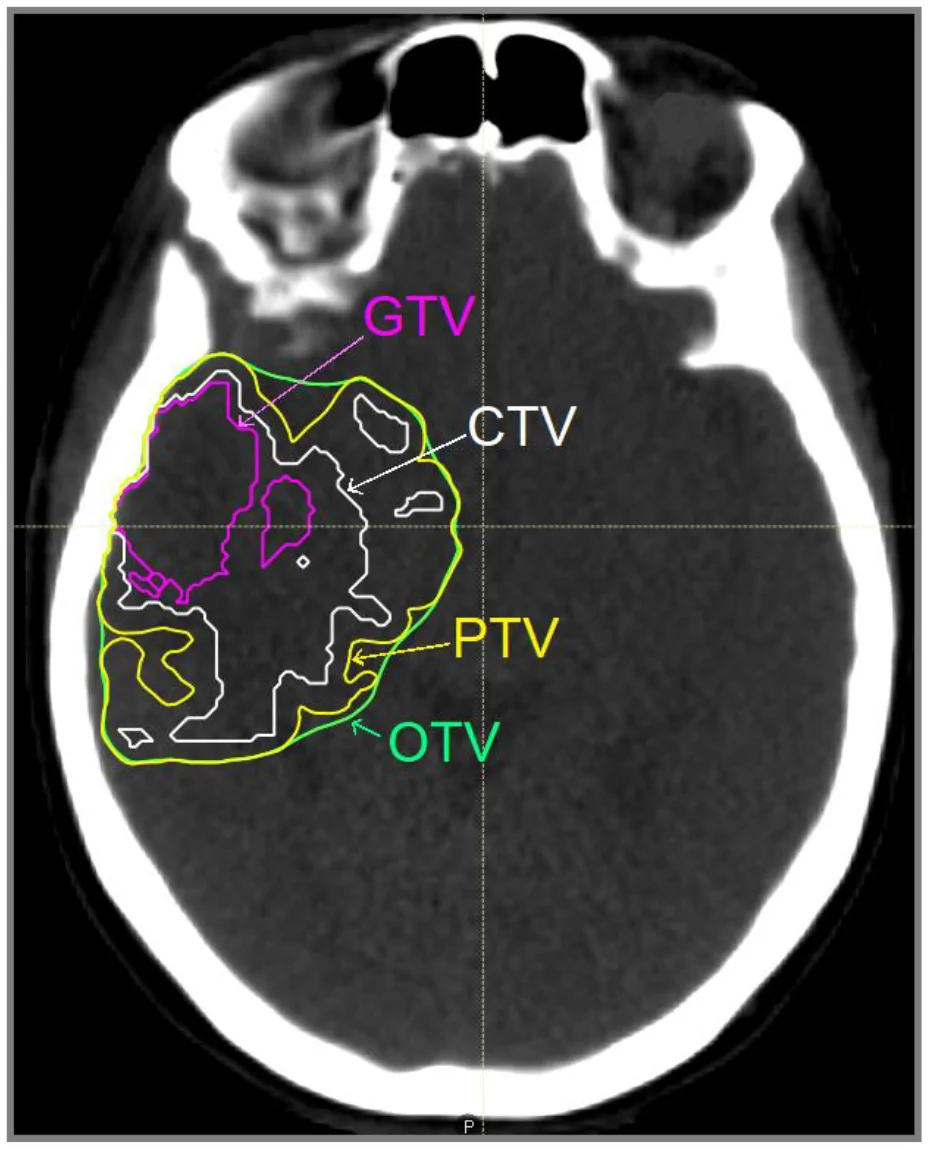
Main targets defined in glioblastoma treatment planning. The case illustrates a highly anisotropic and irregular gross tumor volume (GTV) and predicted tumor spread indicated as the clinical target volume (CTV). To address these complexities and to facilitate treatment planning, it was necessary to construct an optimization treatment volume (OTV) from the planning target volume (PTV).

The anisotropic geometry of the PTVs may complicate finding an optimal solution during plan optimization. When necessary, to simplify the optimization process, optimization target volumes (OTV_1000_, OTV_100_, OTV_10_) were defined by expanding, and subsequently contracting, the PTV margins by 10 mm, equivalent to a morphological closing operation (**Figure 2**).

Both the PTV and OTV were used as treatment volumes when determining the dose to the healthy brain. The EORTC recommends that at least 95% of the PTV is encompassed by the 95% isodose (57.0 Gy) and that no more than 2% of the PTV should be subjected to 107% isodose (64.2 Gy). These restraints and OAR dose constraints were used as clinical goals for evaluating the dose plans. Additionally, the homogeneity index (HI) and conformity index (CI) were evaluated using **Equations 2**, **3**:

(2) $$\text{HI}=\frac{D\left(x\right)}{D\left(100-x\right)}$$

(3) $$\text{CI}=\frac{\text{target volume covered by isodose}}{\text{total isodose volume}}$$

where *D*(*x*) is dose at *x*% volume, and values of 1 correspond to perfect dose homogeneity and conformity, respectively. HI was evaluated at *x* = 98% (HI_98%_), and CI was evaluated at 60 Gy (CI_60 Gy_).

#### Methodology II: voxel-based dose-painting

The methodology described above closely aligns with conventional treatment planning approaches except for the CTV delineation process. However, when a tumor infiltration model (such as the one employed in this study) is available, an alternative strategy may be considered through the use of a clinical target distribution (CTD). Unlike manually delineated targets based on visual assessment from medical imaging, the CTD provides a complete, theoretical cell density distribution throughout the brain at the voxel level. The availability of this distribution enables voxel-based dose-painting strategies, which allow for radiation dose modulation according to the predicted tumor cell density in each voxel (25).

The radiobiologically optimal dose for each voxel can be derived using established mathematical models. The relationship between the surviving fraction of tumor cells and the absorbed dose—specific to a given radiation quality—can be characterized *in vitro*, and is commonly described by the linear-quadratic (LQ) model (26). A dose prescription may be evaluated in terms of tumor control probability (TCP) as a function of absorbed dose to the tumor. Given a known cell density distribution, the dose to each voxel to achieve a predefined TCP can be calculated. In a voxel *i* the local tumor control probability, TCP_i_, is given by **Equation 4**:

(4) $${\text{TCP}}_{i}=e^{-N_{0,i}\text{SF}}$$

where *N*_0,_*_i_* is the number of initial cells in voxel *i*, and SF*_i_* is the surviving fraction of tumor cells in voxel *i* after irradiation. Using the LQ-model as basis for SF, we get

(5) $${\text{TCP}}_{i}=e^{-N_{0,i}e^{-n\left(\alpha d_{i}+\beta d_{i}^{2}\right)}}$$

where *n* is the number of fractions, *d_i_* is absorbed dose per fraction in voxel *i* (Gy), and *α* (Gy^-1^) and *β* (Gy^-2^) are model parameters where the ratio *α*/*β* (Gy) indicates the tissue sensitivity to fractionated radiation schedules. The GBM cell *α*/*β* ratio used in this study was *α*/*β* = 8 Gy (27). The TCP is determined by the product of TCP*_i_* for all voxels *i*:

(6) $$\text{TCP}=\;\prod_{i=1}^{N}{\text{TCP}}_{i}$$

where *N* is the total number of voxels. From Equations 5 and 6, it follows that, for a desired TCP, the required dose to voxel *i* is determined by

(7) $$d_{i}\left(\text{TCP},N_{0,i},N,n,\alpha ,\beta \right)=-\frac{\alpha }{2\beta }\pm \sqrt{{\left(\frac{\alpha }{2\beta }\right)}^{2}+\frac{1}{n\beta }\ln\left(-\frac{N\cdot N_{0,i}}{\ln\left(\text{TCP}\right)}\right)}$$

where only the positive solution is physically relevant. Calculating *d_i_* for each voxel i ∈{1, 2,…,N} yields a dose distribution in the form of a matrix **D** as consisting of TCP-dependent dose elements *d_i_*(TCP). The matrix **D** represents the theoretical optimal dose distribution for a given CTD and desired TCP. In this study, TCP was set to 0.95, and voxels with a cell density less than 1 cell/mm^3^ set to zero.

To facilitate the treatment planning process, fictional targets (*V*_x Gy_) based on the **D** dose isocontours of 80 Gy (*V*_80 Gy_), 60 Gy (*V*_60 Gy_), 40 Gy (*V*_40 Gy_) and 20 Gy (*V*_20 Gy_), were defined. These dose thresholds (*D*_thr_) dictate the minimum relevant dose to be mimicked in the new plan (i.e., the optimizer focused on matching the new dose plan to **D** for *d_i_* ≥ *D*_thr._, while disregarding voxels with *d_i_*< *D*_thr_). By adjusting the weights in the objective functions of *V*_x Gy_, higher dose mimicking accuracy could be achieved for critical high-dose regions, such as the GTV, while giving less priority to lower-dose regions.

Comparison between the optimal dose distributions **D** and the mimicked dose plans was conducted, and differences quantified in terms of quality index (28) using **Equation 8**:

(8) $$Q_{i}=\frac{{\text{(Mimicked dose)}}_{i}}{{\text{(Optimal dose)}}_{i}}=\frac{\delta_{i}}{d_{i}}$$

where *δ_i_* is the mimicked dose plan, and *d_i_* is the theoretical optimal dose given by Equation 7, in voxel *i*, where *d_i_* > 0 Gy. The quality index *Q_i_* can be plotted in a cumulative quality volume histogram (QVH), where a step function at *Q* = 1 corresponds to a perfect match between the mimicked and planned dose distributions. Quality index was quantified as *Q*_0.95–1.07_, defined as the percentage of the mimicked dose plan receiving 95% to 107% of the prescribed reference dose. The quality index was evaluated for the segmented GTV, the conventional clinical PTV (GTV with a 15 mm + 3 mm margin added), and for the whole patient. The volume of healthy brain receiving 60 Gy or more was evaluated, where the healthy brain was defined as the whole brain with the reference dose subtracted.

## Results

The tumor locations of the cohort were distributed as: temporal lobe (30.6%), parietal lobe (29.3%), frontal lobe (26.8%), occipital lobe (12.1%), with two additional cases (1.3%) of ambiguous tumor location. The mean volume of the GTVs of the entire cohort was 31.7 cm^3^, whereas the tumor volumes and volume ratios (GTV_sim_/GTV) of the selected cases were: Case A: 24.2 cm^3^ (1.35); Case B: 41.9 cm^3^ (2.24); Case C: 14.5 cm^3^ (1.81). Comparably, the predicted tumor spread indicated by the volumes of CTV_1000_ were: Case A: 57.74 cm^3^; Case B: 122.7 cm^3^; Case C: 42.8 cm^3^. Observed tumor progression was reported for all three cases at 176 days, 108 days, and 199 days post-radiotherapy, for Case A, Case B, and Case C, respectively.

**Figure 3:**
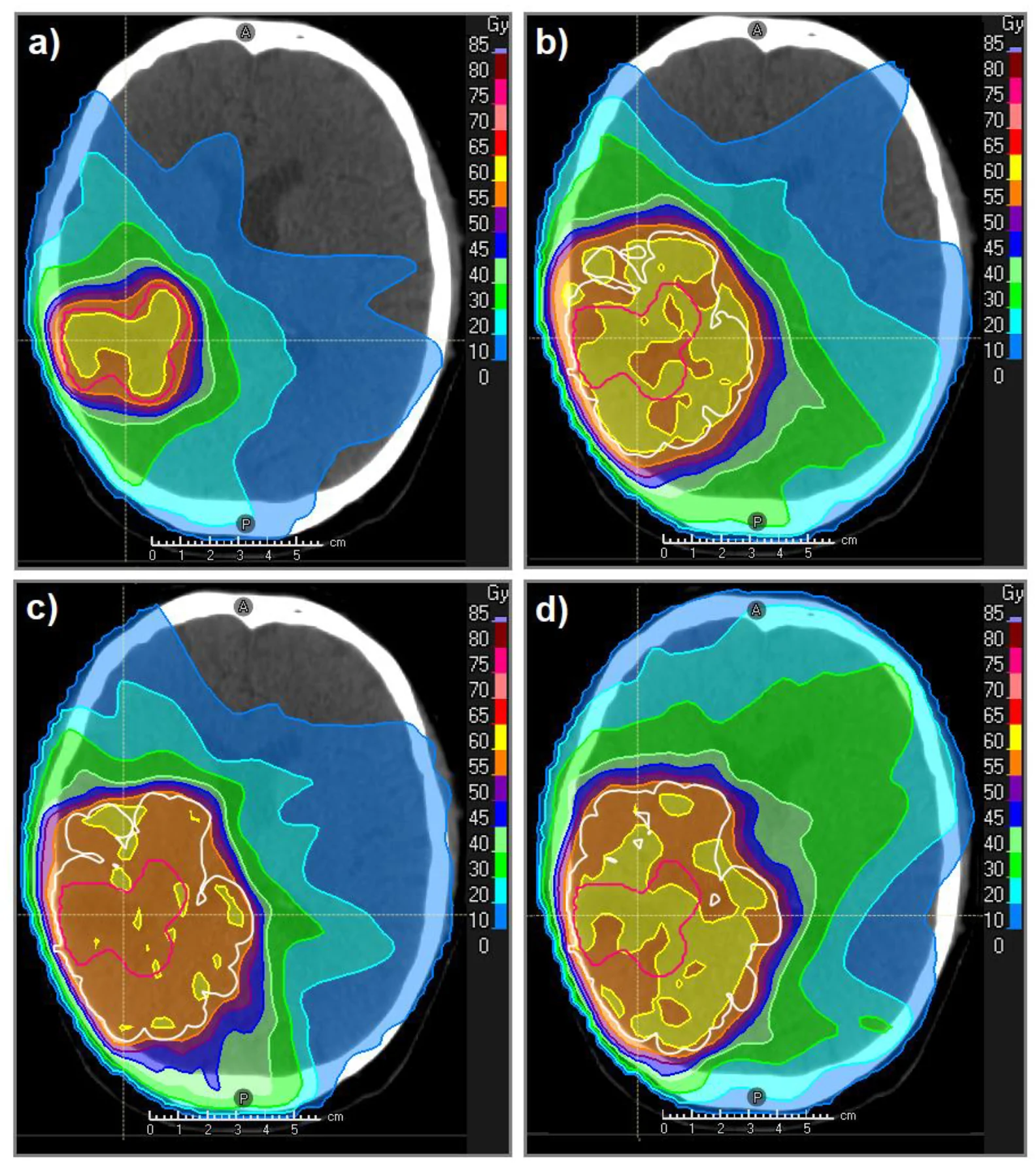
Dose plans of Case B, when treatment plans are optimized for **(a)** the GTV; **(b)** PTV_1000_; **(c)** PTV_100_; and **(d)** PTV_10_. The GTV corresponds to the magenta contour, the PTVs correspond to the white contours, and the colorwash illustrates the dose distributions (Gy). The prescribed dose was 60 Gy (yellow color) for all cases. All treatment plans passed the clinical goals in terms of target coverage and constraints for the organs at risk.

**Figure 4:**
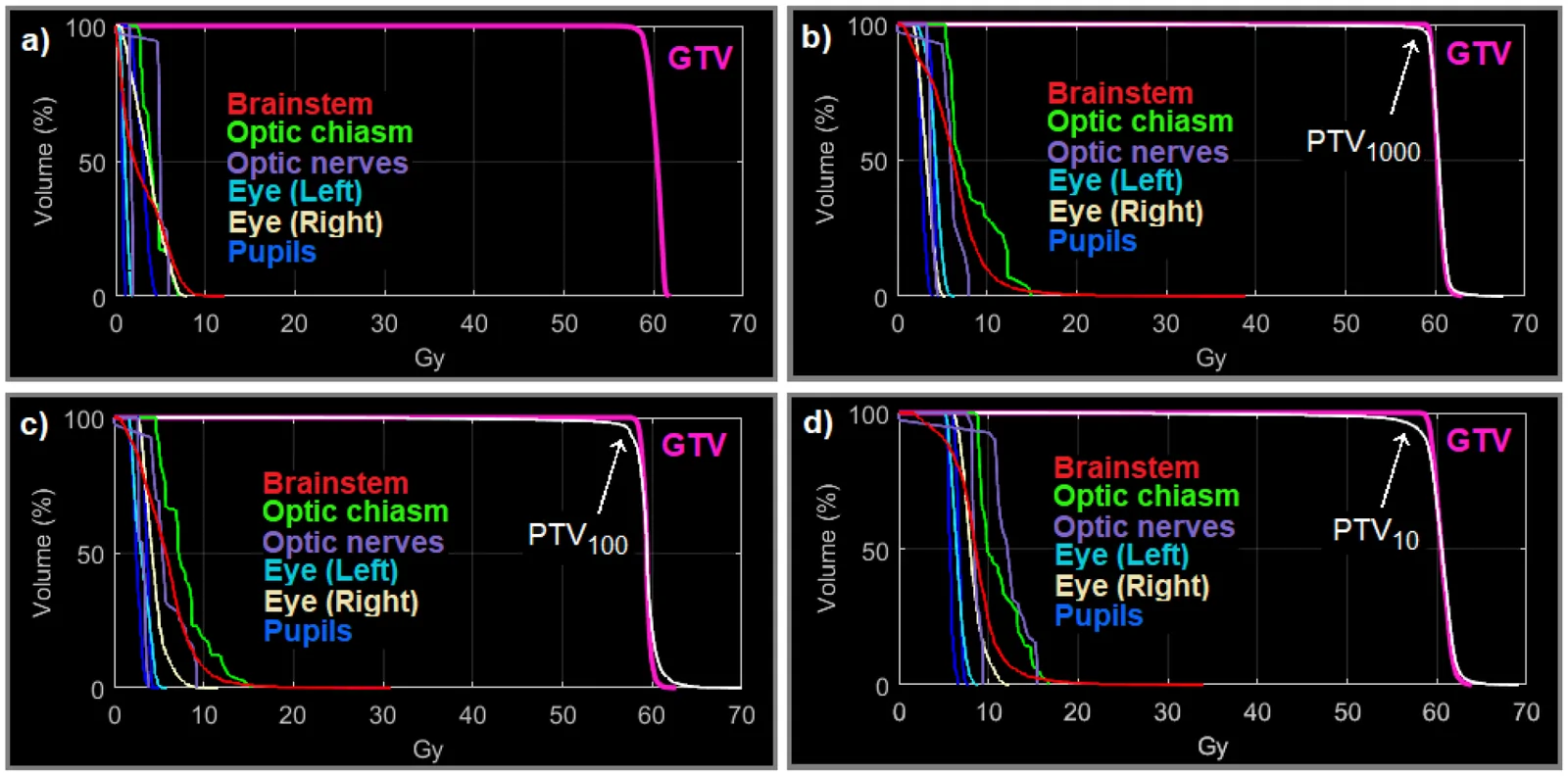
Dose volume histograms (DVHs) of Case B, when treatment plans are optimized for **(a)** the GTV; **(b)** PTV_1000_; **(c)** PTV_100_; and **(d)** PTV_10_. The prescribed dose was 60 Gy for all cases. All treatment plans passed the clinical goals in terms of target coverage and constraints for the organs at risk.

**Figures 3** and **4** show the planned dose distributions the corresponding dose volume histograms (DVHs) for Case B, where treatment plans are optimized for the segmented GTV, PTV_1000_, PTV_100_, and PTV_10_ with a prescription dose of 60 Gy in 30 fractions. *D*_95%_ > 95% isodose and *D*_2%_< 107% isodose were achieved for all dose plans, and doses to OARs were well below the recommended dose limits. Neither HI_98%_ nor CI_60 Gy_ showed a clear correlation with either target size or tumor location. Among all cases and treatment plans, the minimum and mean of the homogeneity and conformity indices were: minimum HI_98%_ = 0.82; mean HI_98%_ = 0.90; minimum CI_60 Gy_ = 0.90; mean CI_60 Gy_ = 0.93. Detailed dose statistics, conformity and homogeneity indexes are reported for all cases in the **Supplementary Data**.

**Figure 5:**
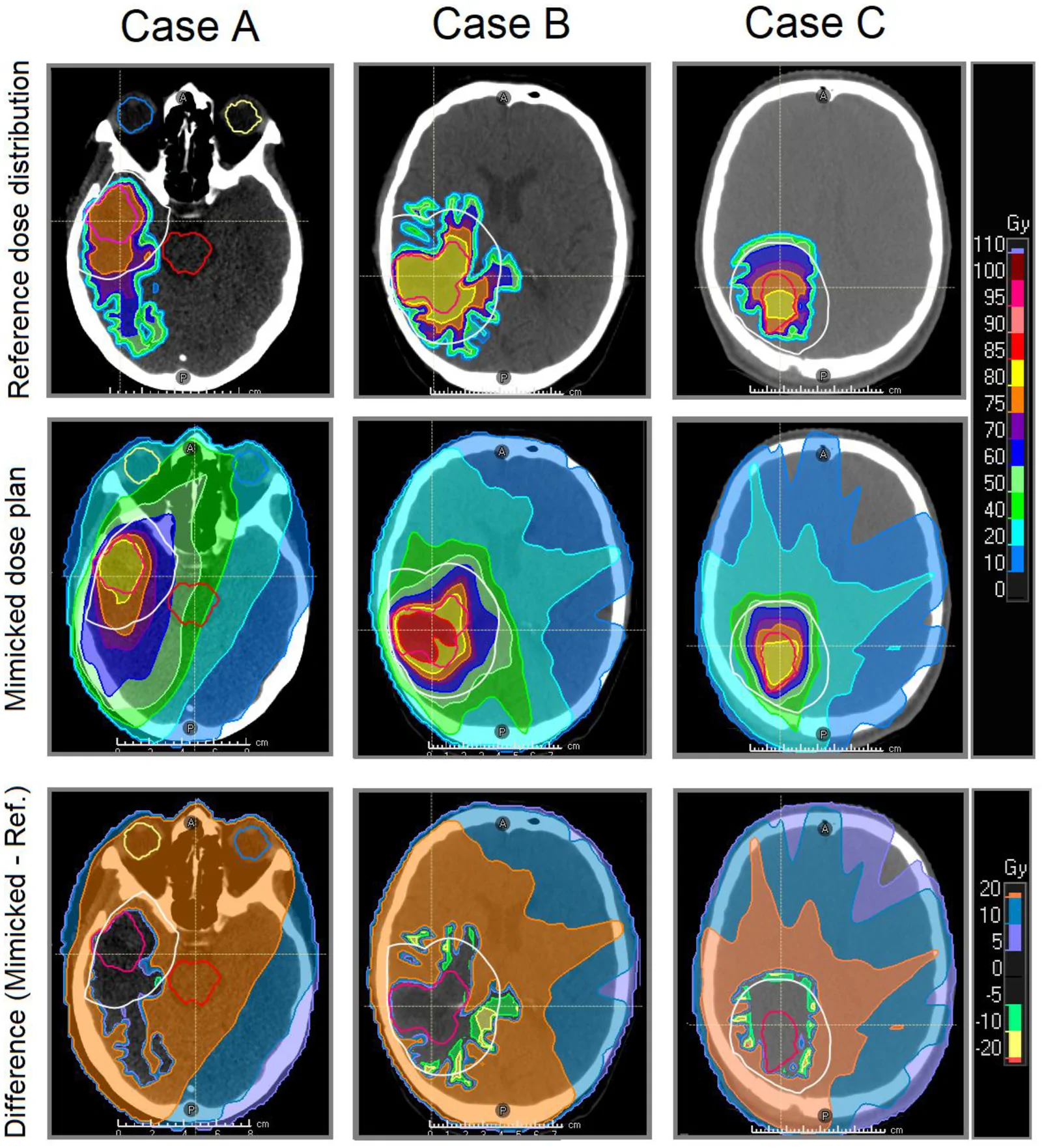
Reference dose distribution, mimicked dose plan, and difference between the two, for all cases. The gross tumor volume (GTV) corresponds to the magenta contour, and the planning target volume (PTV) corresponds to the white contour. In Case A, the brainstem and eyes are delineated with red, blue, and yellow contours, respectively. Considerable irradiation of healthy brain tissue is, however, evident.

**Figure 5** shows the reference dose distributions, the corresponding mimicked dose plans, and the resulting differences between them, for all cases. Adequate replication of the dose reference distributions was achieved by the mimicked dose plans within the segmented GTV, although a few hot spots with doses exceeding the reference dose by 5 Gy, as well as cold spots with doses 10 Gy less than the reference dose, were observed. More pronounced differences emerged when the PTV and whole brain were considered as targets. In particular, the mimicked plans result in the unavoidable irradiation of healthy tissue, highlighting the trade-off between dose conformity and normal tissue sparing at larger target volumes.

**Figure 6:**
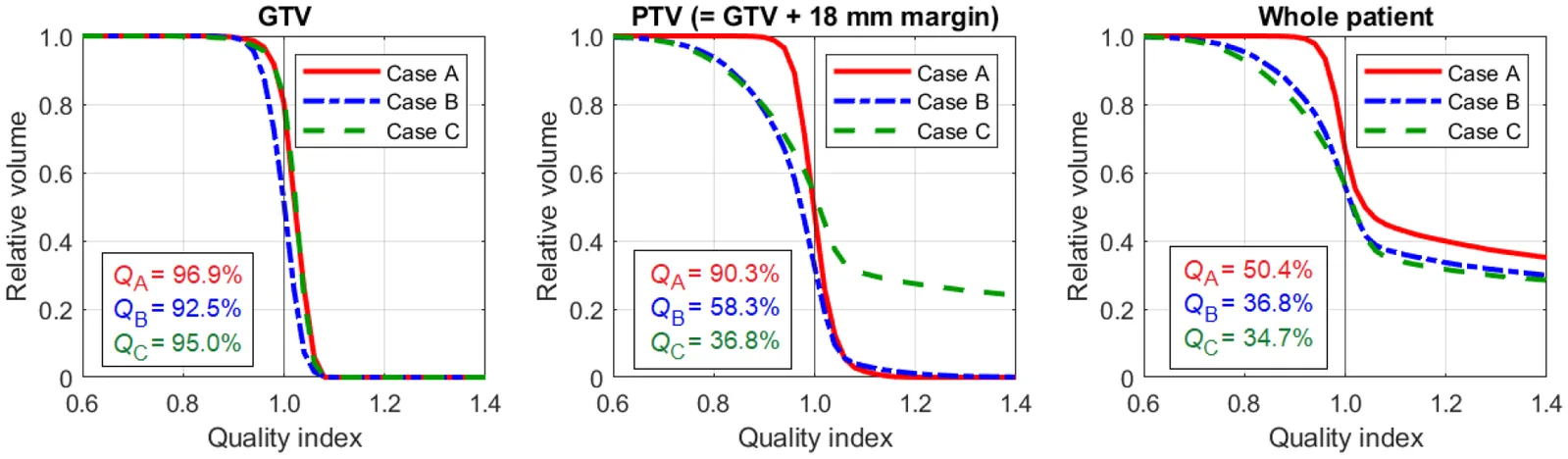
Quality volume histograms (QVHs) of the three cases, evaluated within the GTV, the clinical PTV (defined as the GTV with an added 18 mm margin), and for the whole brain. *Q*_A_, *Q*_B_, and *Q*_C_ represent *Q*_0.95-1.07_ for Case A, B, and C, respectively.

**Figure 6** shows the QVHs for all cases and target volumes. The plan quality decreases with increasing target volume, as evidenced by the decrease of *Q*_0.95–1.07_ across all cases. The closest agreement between the mimicked dose plan and reference dose distribution was seen for Case A for the GTV, with Q_0.95–1.07_ = 96.9%. In contrast, the poorest agreement occurred in Case C when evaluating the whole patient, with *Q*_0.95–1.07_ = 34.7%.

The volumes of healthy tissue receiving a dose of 60 Gy or more were 60.61 cm^3^, 4.84 cm^3^, and 0.28 cm^3^, for Case A, B, and C, respectively, indicating varying degrees of dose spillage into normal brain tissue depending on tumor location and extent.

## Discussion

Efficient treatment for GBMs remains a significant challenge. The high recurrence rates following treatment have prompted reconsideration of target volume delineation, leading to changes in isotropic CTV margin widths. However, despite these changes, no improvement in overall survival has been correlated with increased CTV margins. In fact, the latest EORTC recommendations even suggested a 5 mm margin decrease to reduce side effects such as radiotoxicity, as overall survival remains unchanged. This suggests that while enhancing treatment efficacy is crucial, efforts to significantly improve GBM survival using isotropic CTVs may have reached their limits. The debate over CTV shape remains unresolved due to insufficient clinical evidence on infiltrated tissue, as conventional medical imaging often lacks the resolution to accurately delineate these regions.

Identifying and localizing normal tissue infiltrated by GBM cells remains a key issue in treating GBMs. Models have been developed to predict tumor spread *in silico* (17, 18, 20, 24, 29, 30*).* The conventional assumption is that GBMs are composed of solid tumors with an isotropic cell invasion. The EORTC recommends prescribing the same dose to the GTV as to the CTV, implying that the cell density is equal in both regions. On the contrary, models predict that the tumor cells show a preference for spread via the white matter, resulting in anisotropic cell distributions. Additionally, the cell density decreases approximately exponentially from the GTV edge. A fundamental limitation of glioma tumor models is how to verify their accuracy with respect to tumor cell distribution, since the localization of individual tumor cells from image data alone is impossible. However, the model used in this study has been evaluated in terms of predicted tumor cell distribution pre- and post-treatment, which have been shown to correlate with overall survival (21), and early treatment outcome (22). These results provide a basis for further investigation of the proposed model, both in terms of additional model verification, and practical clinical consequences. This study presents two methodologies for dose planning on anisotropic targets, tumor cell density distributions derived from modeling.

Tumor spread models exhibit a wide range of complexity, but generally assume the same fundamental principles: tumor cell motility is greater in white matter than in gray matter, tumor cells may not cross anatomical barriers, the cell density is limited by an intrinsic normal tissue tumor cell carrying capacity, and the change of cell density with time is governed by cell proliferation, cell diffusion, and cell killing by treatment. The model used in this study was based on a commonly used Fisher-Kolmogorov diffusion-proliferation model (23, 24, 31). The characteristics of a tumor in terms of normal tissue invasion aggressiveness and tumor spread isotropy can be described by the ratios of *D_W/_ρ* and *D_W_*/*D_G_*, respectively, where *D_W_* is the diffusion coefficient in white matter, *ρ* is the proliferation constant, and *D_G_* is the diffusion coefficient in gray matter. Due to the heterogeneous nature of GBMs and interpatient variability, input parameters of *D/ρ* and *D_W_* > *D_G_* reported and used in the literature cover a large range of values (23, 24, 29, 32), which were covered in the simulations in this study. Despite this variability, the assumption that *D_W_* > *D_G_* results in anisotropic tumor spread holds true regardless of the specific ratio value. Conventional isotropic CTV expansion for high-grade gliomas has been questioned due to differences in cell motility (20). While the parameter D/ρ influences the magnitude of tumor spread, it does not affect the direction of spread, as the white-to-gray matter diffusion coefficient ratio remains constant regardless. In practice, this uncertainty translates into variability in the cell density threshold value used for CTV delineation, analogous to the current uncertainties in conventional CTV margins. While there are uncertainties associated with the normal tissue’s tumor-carrying capacity and the initial number of tumor cells seeded for simulation, the model has demonstrated high robustness with respect to these parameters (20).

The progression status of all three selected cases was known at the time of analysis, allowing for the investigation of treatment planning strategies in scenarios where early progression had been reported and where alternative planning methodologies might have the greatest potential impact. These cases were deliberately chosen to represent the broader cohort in terms of tumor size and anatomical location. In the entire cohort, the most common tumor locations were the temporal lobe (30.6%) and the parietal lobe (29.3%), which was represented in the selected cases. A key inclusion criterion was documented post-treatment tumor progression, under the rationale that alternative treatment planning approaches may be the most impactful where the treatment provided may have been suboptimal. To capture a range of planning challenges, variation was introduced through case selection: Case A was chosen because of the predicted tumor spread’s vicinity to the brainstem, Case C was characterized by a tumor located far from OARs and major anatomical barriers, representing a less constrained planning scenario, and Case B was chosen as an intermediate case in terms of anatomical complexity and planning difficulty. Consequently, the methodology used in treatment planning for the three selected cases is largely transferable to most variations of single-focal GBMs with respect to tumor size and location, and may be used for additional investigation on more selectively curated datasets.

When optimizing on OTVs, the prescribed dose may include apparent healthy tissue as defined by the PTV. Neglecting the healthy tissue between infiltrated tissue regions may be necessary to ensure acceptable dose coverage to the CTVs. Clearly, this is dependent on the geometry of the CTV and the dose delivery precision of the irradiation modality. In this study, an OTV was created using morphological closing with a 10 mm radius, effectively removing any gaps smaller than 20 mm in the infiltrated region. While this width was arbitrarily selected to ensure plan robustness, future work may explore smaller closing radii depending on anatomical constraints and delivery precision. The EORTC recommends that a PTV margin of 3 mm be added to the CTV to account for uncertainties in the clinic caused by limitations in dose delivery accuracy and patient fixation. While a 3 mm margin is generally adequate, it may be insufficient to address gaps caused by the distance of white matter between brain gyri, as predicted by anisotropic tumor spread modeling. The morphological closing operation applied to OTVs makes them resemble conventional CTV delineations. However, the targets are not directly comparable since the OTV is defined based on cell infiltration rather than an arbitrary margin width, resulting in a target that is not necessarily isotropic but instead reflects the direction of tumor spread.

For GBMs, it is reasonable to consider that ensuring adequate coverage of the CTV is of greater concern than sparing surrounding healthy tissue, given the persistent challenges in achieving a high TCP clinically. The healthy tissue in these regions may also be considered of lower priority, as ensuring adequate CTV coverage will inevitably lead to irradiation of these regions with doses of similar magnitude, thereby sparing only a few healthy cells. Nonetheless, it should be noted that the CTV should still be considered representative of the true target, and coverage of the PTV should be clinically evaluated when dose planning. Should more conformal radiotherapy modalities be available, efforts should be made to reduce the closing width and PTV margin. In this study, the dose prescribed to the PTVs was 60 Gy. The isocontours of 1000 cells/mm^3^, 100 cells/mm^3^, and 10 cells/mm^3^ are predicted to incorporate cell densities smaller than the cell density in the GTV, suggesting that a lower dose is needed in the CTV to achieve the same TCP as in the GTV. Therefore, an alternative approach could involve lowering the prescribed dose towards the lower cell density isocontours. However, prescribing a dose for regions with lower cell densities is challenging, as it relies on radiobiological models rather than empirical evidence. Disregarding uncertainties in the cell density values themselves, the calculation of effective cell killing from irradiation relies on radiobiological models rather than empirical evidence. The recommended dose of 60 Gy to the CTV, as used in the Stupp protocol (1), is based on clinical evidence rather than theoretical models. Barring more clinical evidence of TCP as a function of dose in CTVs, adjustments to prescribed dose are unlikely to be implemented (especially if a lower dose is recommended). Consequently, in accordance with the recommendations from the EORTC, the doses were kept constant at 60 Gy for the GTV and PTV when using the anisotropic CTV methodology.

The Radiation Therapy Oncology Group (RTOG) recommends a boost-down method (33), based on the assumption that T1 shows the GTV and T2 the diffusive spread (34). While it is agreed that the gross tumor exhibits a hyperintense signal on T1 imaging, the relevance of T2 and FLAIR sequences for imaging tumor tissue is inconclusive. Even so, T2 and FLAIR sequencing have been used to determine the extent of diffusive GBM cell invasion (35–38). While such sequences may decrease the visibility threshold to allow detection of cell densities less than 8000 cells/mm^3^, they also highlight other tissues and fluids, such as the fluid present in edema. A correlation between tumor cell invasion and edema has been suggested, but remains uncertain (6, 39–41). For this reason, the RTOG recommends including edema in the CTV delineation, whereas the EORTC does not. T2 and FLAIR images were available in this study but were not considered for **Supplementary Data** on the tumor spread, due to the additional uncertainties they may introduce and the lack of consensus on their relevance for this purpose.

The mimicked dose plans showed better agreement with the voxel-based reference dose distributions within the GTV delineation. The reference dose distributions exhibited steep dose gradients where anatomical barriers impeded a smooth and continuous decrease in tumor cell density during tumor spread simulation. The mimicked dose plans consequently administered overdosage to the edges of the reference dose distribution since target coverage was prioritized over a sharp dose fall-off in normal tissue. While the GTV was primarily located in the central region of the reference dose distribution, thus avoiding the edges of the reference distribution, the successive larger delineations of the PTV and the whole brain increased the surface area included in the delineations rendering poorer agreement between plans. Care must, therefore, be taken when mimicking dose plans on reference distributions that are highly anisotropic, as the increased surface area included in the delineation may be difficult to replicate. Additionally, the mimicked dose plans unavoidably include irradiation of healthy tissue due to the beam entrance and exit points, which combined with highly irregular tumor shapes, may lead to large doses to the healthy brain. For further investigation, Gaussian smoothing of the reference dose distributions along edges may be justified, as may the reconsideration of other targets wherein to determine *Q*_0.95-1.07_. The quality index, however, remains a useful tool for determining the replication accuracy between two mimicked plans for the same reference dose distribution and target.

Currently, *in vivo* determination of cell densities and their distribution for GBM cases is not feasible, and sufficient dose coverage is preferred. In this study, isocontours of cell densities lower than 10 cells/mm^3^ were not considered for dose planning, despite being available from tumor simulations. As the cell density isocontour approaches zero, the encompassed volume rapidly increases, which would result in irradiation of 60 Gy to considerably large volumes of apparently healthy tissue.

Voxel-based dose painting addresses the challenges associated with CTV definition by allowing the dose to be tailored based on tumor cell count. An additional feature of this methodology is its capacity to prescribe doses exceeding 60 Gy to the inner core of the GTV. The predicted cell density determined by the tumor spread model used is limited by the normal tissue tumor cell carrying capacity, set to 2.39 x 10^5^ cells/mm^3^ (42). Voxels in the inner GTV core frequently reached this limit. It is seen from Equation 7 that 60 Gy is not sufficient to achieve a TCP ≥ 0.95, as is shown by the reference dose distributions in this study. Although dose escalation studies have not demonstrated clear benefits, these studies applied dose escalation uniformly across the entire GTV (43–45). Other studies have suggested that dose escalation to the tumor inner core may increase TCP, since the frequency of hypoxic cells is likely to increase towards the tumor center-of-mass (46–48). Conversely, the dose to the GTV could easily be artificially limited to 60 Gy to more closely resemble current oncological protocols, potentially easing the adaptation of this strategy into clinical practice.

Despite potential limitations in dose delivery, voxel-based dose-painting remains uncertain due to the reliance on radiobiological modeling to compute the optimal dose for achieving a specified TCP. The LQ-model, while commonly used, is associated with uncertainties in its input parameters, such as the α/β ratio, which introduces variability in dose scaling.

To improve the treatment outcome of GBMs, tumor spread modeling has been thoroughly explored, but the practical implementation of tumor models has been less investigated. A modified solution to the model used in this study has been applied by Unkelbach et al. (29) on ten GBM cases, where theoretical dose plans were compared to their clinical counterparts. The results showed that there was sensitivity to CTV delineation when the tumors were situated near the corpus callosum. Radiosurgical treatment planning for GBMs has also been investigated by Lazzeroni et al. (49) who showed that plans targeting the GTV were inadequate when tumor spread was assumed, and that treatment plans could potentially be improved if prior knowledge of tumor spread was available.

The three cases considered for treatment planning in this study had a reported follow-up status of tumor recurrence. Alternative treatment methodologies must be explored to potentially reduce both the rate and speed of recurrence. This study evaluated the feasibility of two methodologies for treatment planning based on modeled tumor cell distributions. The first approach, keeping practical considerations in mind by following the current recommendations closely, offers a smooth transition from conventional to cell density-based treatment planning. The second approach, which pushes treatment planning to its theoretical limits, could potentially drive advancements in GBM treatment toward improved outcomes.

## Conclusion

The poor treatment outcome for GBMs has raised questions about the current CTV delineation practices and how to adapt a novel definition of the CTV into practical treatment planning. This study simulated tumor spread in three GBM cases and explored two methodologies for treatment planning on the anisotropic tumor cell distributions. The first methodology adhered closely to current treatment guidelines, whereas the second considered the theoretical optimal dose distribution on a voxel-by-voxel basis. The planning method accounting for the anisotropic CTV delineation rendered clinically acceptable dose distributions. However, for the voxel-based dose distribution method, special attention must be given when mimicking dose distributions of large and anisotropic volumes, since the sharp dose gradients of the reference dose distributions may be difficult to replicate.

## Data Availability

The raw data supporting the conclusions of this article will be made available by the authors, without undue reservation.
