# Supplementary material for: Treating the invisible target: treatment planning targeting the microscopic tumor spread in gliomas
Source: Front Oncol. 2026 Sep 9;16:1872089. doi: 10.3389/fonc.2026.1872089 (PMC13597432; doi:10.3389/fonc.2026.1872089)
Supplement: Supplementary file 1 [file DataSheet1.pdf]

Supplementary Materials

Supplementary Table 1: Dose statistics for dose plans as a function of treatment target for all cases, for the gross tumor volume (GTV) and planning target volumes (PTV_X), where the index X corresponds to the volume delineated by the cell density isocontour of X cells/mm^3. $D_{95\%}$ corresponds to the percentage of target that was covered in at least 95% of the prescribed dose. $D_{2\%}$ corresponds to the percentage of target that was covered in more than 107% of the prescribed dose. The recommended limits are 95% and 2% for $D_{95\%}$ and $D_{2\%}$, respectively. $HI_{98\%}$ is the homogeneity index at 98% volume, and $CI_{60\ Gy}$ is the conformity index at 60 Gy isodose. $V_{Brain\setminus\Omega@60\ Gy}$ is the healthy brain volume (cm^3) receiving 60 Gy or more (recommended to be less than 3 cm^3) where Ω denotes the volume subtracted from the whole brain e.g. $V_{Brain\setminus OTV}$ is the entire volume of the brain not part of the optimization treatment volume (OTV). OTVs were not used for Case C.

		Case A	Case B	Case C
**GTV**	$D_{95\%}$	99.7%	99.7%	99.1%
	$D_{2\%}$	0%	0%	0%
	$HI_{98\%}$	0.83	0.84	0.82
	$CI_{60\ Gy}$	0.90	0.94	0.90
	$V_{Brain\setminus GTV@60\ Gy}$	0.22 cm^3	0.29 cm^3	0.97 cm^3
**PTV_{1000}**	$D_{95\%}$	98.8%	99.1%	99.9%
	$D_{2\%}$	0.27%	0.47%	0%
	$HI_{98\%}$	0.93	0.94	0.97
	$CI_{60\ Gy}$	0.90	0.91	0.95
	$V_{Brain\setminus PTV@60\ Gy}$	2.95 cm^3	4.13 cm^3	0.75 cm^3
	$V_{Brain\setminus OTV@60\ Gy}$	0.51 cm^3	1.83 cm^3	-
**PTV_{100}**	$D_{95\%}$	97.7%	96.8%	99.9%
	$D_{2\%}$	0%	0.61%	0%
	$HI_{98\%}$	0.92	0.89	0.96
	$CI_{60\ Gy}$	0.91	0.93	0.95
	$V_{Brain\setminus PTV@60\ Gy}$	4.28 cm^3	0.99 cm^3	1.12 cm^3
	$V_{Brain\setminus OTV@60\ Gy}$	0.39 cm^3	0.07 cm^3	-
**PTV_{10}**	$D_{95\%}$	96.4%	95.8%	99.6%
	$D_{2\%}$	0%	0.59%	0%
	$HI_{98\%}$	0.89	0.88	0.96
	$CI_{60\ Gy}$	0.93	0.96	0.97
	$V_{Brain\setminus PTV@60\ Gy}$	5.90 cm^3	1.24 cm^3	0.95 cm^3
	$V_{Brain\setminus OTV@60\ Gy}$	0.17 cm^3	0.17 cm^3	-

Supplementary Table 2: For Case A, dose statistics for available organs at risk, for all plans. The index *X* of D_X corresponds to the dose received to *X*% of total organ volume.

		Dose (cGy)						
	**Organ**	**D_{99}**	**D_{98}**	**D_{95}**	**Average**	**D_{50}**	**D_2**	**D_1**
**GTV**	Brainstem	30	38	62	463	477	794	831
	Eye (left)	583	614	649	835	841	1014	1028
	Eye (right)	700	732	748	921	919	1142	1169
**PTV_{1000}**	Brainstem	102	114	161	750	680	1886	2206
	Eye (left)	755	759	814	999	989	1266	1311
	Eye (right)	853	892	934	1209	1202	1532	1566
**PTV_{100}**	Brainstem	161	175	216	886	898	1924	2183
	Eye (left)	1033	1041	1069	1248	1250	1474	1488
	Eye (right)	922	948	978	1196	1194	1466	1489
**PTV_{10}**	Brainstem	188	206	262	983	1060	1807	2060
	Eye (left)	960	974	1019	1209	1187	1500	1560
	Eye (right)	908	933	968	1243	1235	1587	1604

Supplementary Table 3: For Case B, dose statistics for available organs at risk, for all plans. The index *X* of D_X corresponds to the dose received to *X*% of total organ volume.

		Dose (cGy)						
	**Organ**	**D_{99}**	**D_{98}**	**D_{95}**	**Average**	**D_{50}**	**D_2**	**D_1**
**GTV**	Brainstem	5	7	13	300	210	832	869
	Optic chiasm	247	260	272	413	392	690	699
	Eye (left)	35	41	52	106	105	170	177
	Eye (right)	71	77	91	365	362	699	723
	Optic nerve (left)	154	154	154	174	176	192	192
	Optic nerve (right)	0	0	373	489	495	594	594
	Pupil (left)	52	52	54	76	74	113	113
	Pupil (right)	157	162	186	297	398	431	448
**PTV_{1000}**	Brainstem	73	90	131	635	627	1454	1702
	Optic chiasm	541	541	543	833	698	1474	1484
	Eye (left)	232	244	275	421	429	563	576
	Eye (right)	185	196	207	327	323	477	494
	Optic nerve (left)	329	329	329	371	367	444	450
	Optic nerve (right)	0	0	249	590	595	792	792
	Pupil (left)	316	326	345	404	411	448	449
	Pupil (right)	198	293	210	274	266	361	374
**PTV_{100}**	Brainstem	77	93	141	582	575	1332	1522
	Optic chiasm	466	466	470	781	718	1468	1510
	Eye (left)	171	176	186	310	299	485	495
	Eye (right)	274	282	295	445	423	783	838
	Optic nerve (left)	268	268	268	313	320	387	387
	Optic nerve (right)	0	0	206	576	539	923	923
	Pupil (left)	166	167	172	252	247	355	368
	Pupil (right)	281	284	293	363	362	423	426
**PTV_{10}**	Brainstem	83	145	213	1032	1058	1758	1926
	Optic chiasm	796	796	802	1029	898	1576	1603
	Eye (left)	459	490	530	933	909	1400	1427
	Eye (right)	501	522	544	799	771	1267	1319
	Optic nerve (left)	755	757	763	872	866	1062	1069
	Optic nerve (right)	0	0	858	1299	1367	1466	1466
	Pupil (left)	588	641	647	968	1000	1263	1274
	Pupil (right)	465	469	483	560	547	693	741

Supplementary Table 4: For Case C, dose statistics for available organs at risk, for all plans. The index *X* of D_X corresponds to the dose received to *X*% of total organ volume.

	Dose (cGy)							
	**Organ**	**D_{99}**	**D_{98}**	**D_{95}**	**Average**	**D_{50}**	**D_2**	**D_1**
**GTV**	Brainstem	0	0	0	15	5	71	81
	Optic chiasm	17	17	17	26	26	43	43
	Eye (left)	2	2	2	6	6	13	13
	Eye (right)	2	2	4	14	13	29	31
	Optic nerve (left)	6	6	7	8	8	11	11
	Optic nerve (right)	15	15	15	19	21	22	23
	Pupil (left)	3	3	4	6	6	10	10
	Pupil (right)	4	4	4	10	10	16	16
**PTV_{1000}**	Brainstem	0	0	3	219	99	1113	1229
	Optic chiasm	162	169	206	347	286	753	753
	Eye (left)	18	19	21	57	47	149	155
	Eye (right)	32	34	38	106	79	316	338
	Optic nerve (left)	50	50	51	71	73	95	95
	Optic nerve (right)	122	123	126	222	213	302	308
	Pupil (left)	26	26	29	53	47	115	117
	Pupil (right)	35	36	38	62	57	109	110
**PTV_{100}**	Brainstem	1	7	25	339	189	1424	1573
	Optic chiasm	192	198	218	389	384	812	812
	Eye (left)	28	29	33	64	60	118	120
	Eye (right)	73	81	98	274	258	539	556
	Optic nerve (left)	66	66	69	86	86	108	110
	Optic nerve (right)	385	386	392	534	526	635	644
	Pupil (left)	38	39	41	60	57	100	101
	Pupil (right)	90	94	103	197	193	326	331
**PTV_{10}**	Brainstem	22	35	58	416	282	1409	1620
	Optic chiasm	229	237	253	397	399	719	719
	Eye (left)	34	35	40	76	69	158	162
	Eye (right)	140	149	223	421	429	639	654
	Optic nerve (left)	86	86	89	114	114	141	142
	Optic nerve (right)	494	495	505	573	587	643	652
	Pupil (left)	47	47	50	64	61	88	90
	Pupil (right)	211	217	229	361	379	466	467

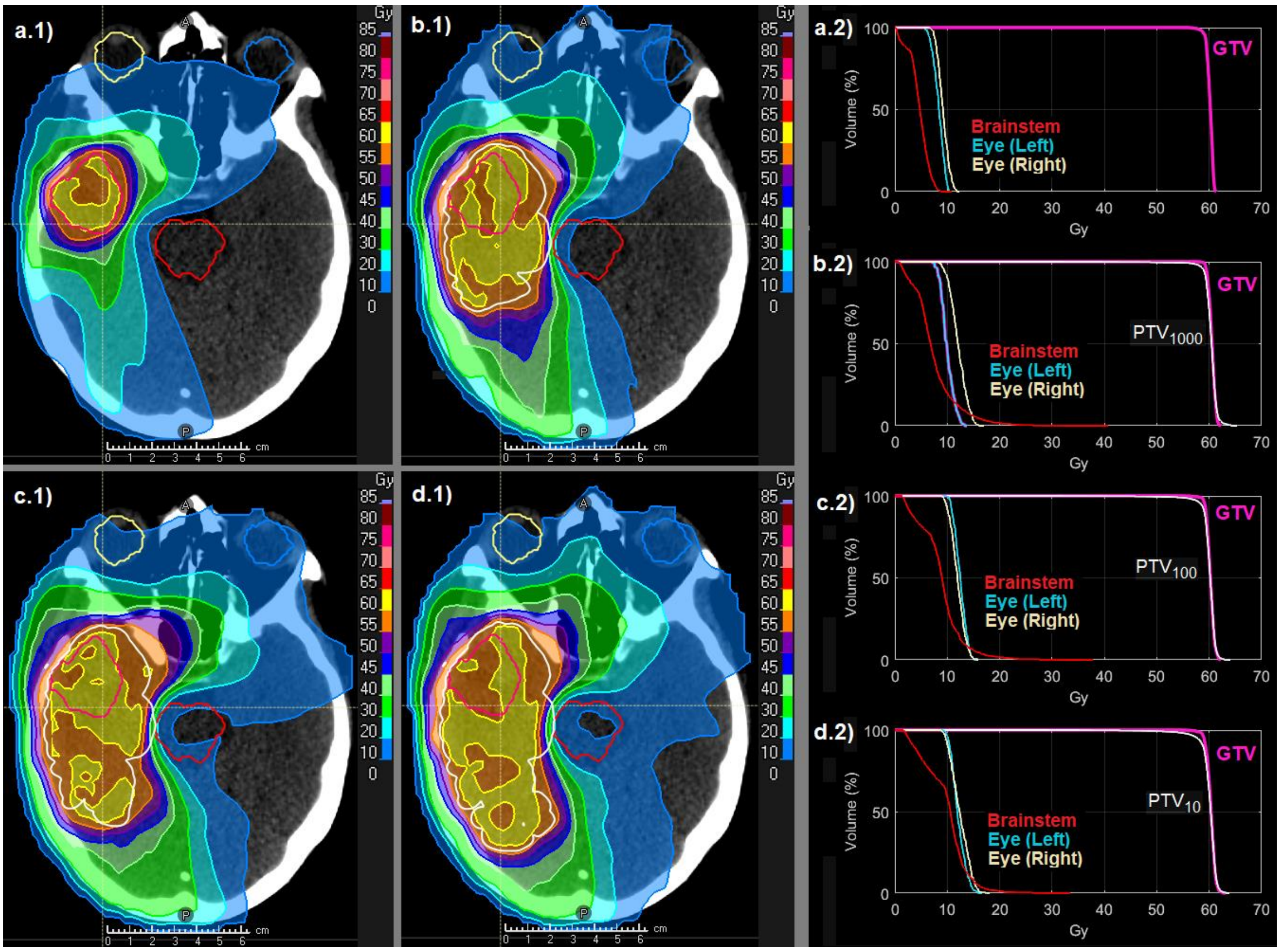

Supplementary Figure 1: Dose plans (x.1) and corresponding dose volume histograms (DVHs) (x.2) of Case A, when treatment plans are optimized for a) the GTV; b) PTV_{1000}; c) PTV_{100}; and d) PTV_{10}. The GTV corresponds to the magenta contour, the PTVs correspond to the white contours, and the colorwash illustrates the dose distributions (Gy). All treatment plans passed the clinical goals in terms of target coverage and constraints for the organs at risk.

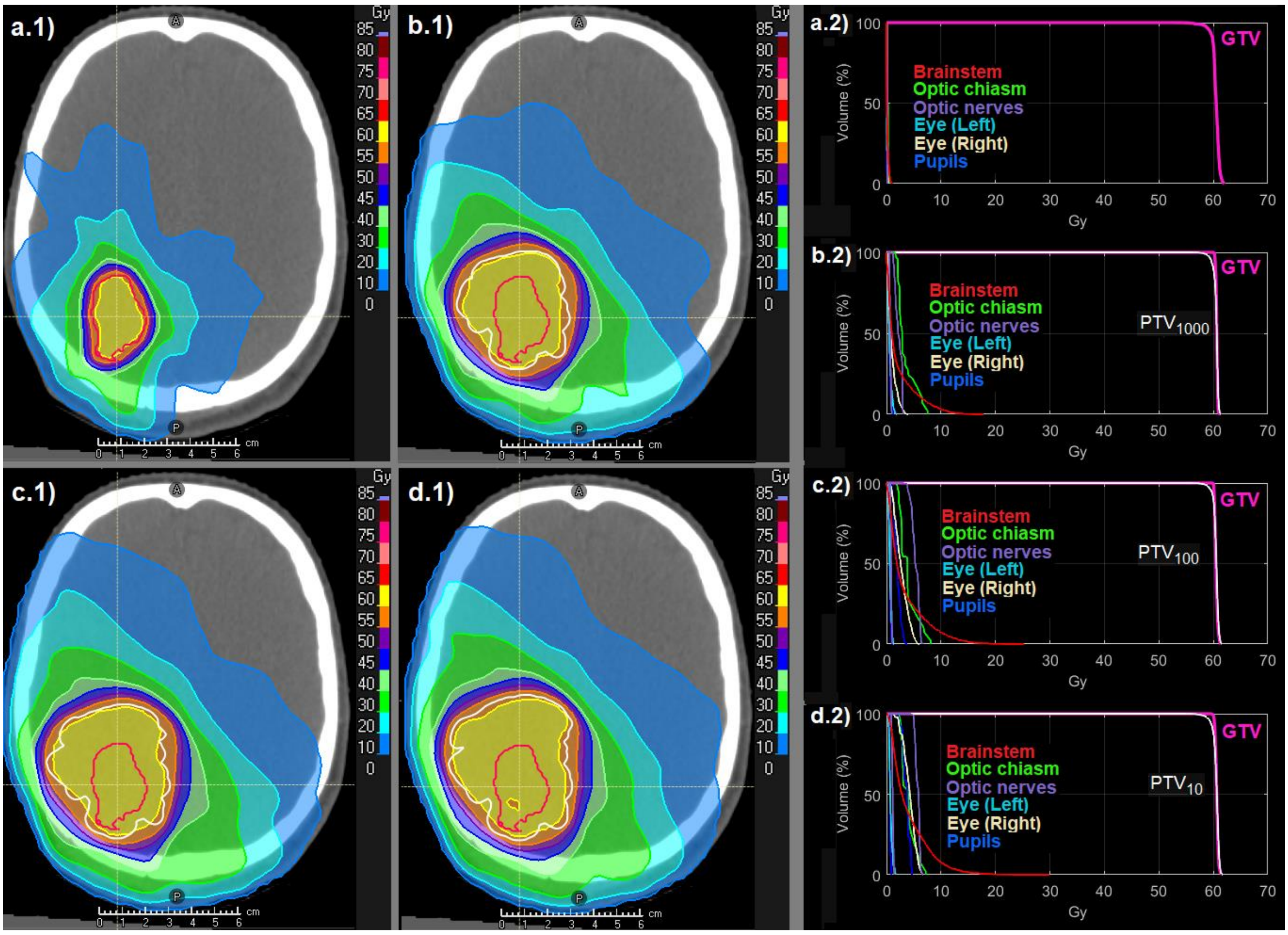

Supplementary Figure 2: Dose plans (x.1) and corresponding dose volume histograms (DVHs) (x.2) of Case C, when treatment plans are optimized for a) the GTV; b) PTV_{1000}; c) PTV_{100}; and d) PTV_{10}. The GTV corresponds to the magenta contour, the PTVs correspond to the white contours, and the colorwash illustrates the dose distributions (Gy). All treatment plans passed the clinical goals in terms of target coverage and constraints for the organs at risk.
